# Pediatric primary diffuse leptomeningeal melanomatosis: Case report and review of the literature

**DOI:** 10.1097/MD.0000000000019178

**Published:** 2020-02-21

**Authors:** Xinke Xu, Yaqi Zheng, Junliang Li, Fenghua Wang, Fangcheng Li

**Affiliations:** a Department of Neurosurgery; b Department of Operating Room; c Department of Pathology, Guangzhou Women and Children's Medical Center, Jin Shui Lu, Guangzhou, China.

**Keywords:** melanoma, melanomatosis, pediatric, leptomeninges, central nerve system

## Abstract

**Rationale:**

Primary melanocytic tumors of central nerve system (CNS) are rare, primary diffuse leptomeningeal melanomatosis (PDLM), a subtype of malignant melanomas of CNS, is extremely rare,especially in pediatrics. As the clinical manifestation of PDLM is not characteristic, It is often misdiagnosed as tubercular meningitis and hemorrhage.

**Patient concerns:**

A 13-year-old boy was admitted to our department with symptoms of recurrent headache and vomiting twice. As the brain imaging revealed a lesion located in the left temporal lobe mimicked hemorrhage, so there was a misdiagnosis of hemorrhage in first hospitalization. He was admitted again for the recurrence of the headache and vomiting. Detailed physical examination showed multiple melanin changes in the skin of the whole body which were ignored in last hospitalization. Brain imaging showed the significantly enlarged lesion in the left temporal lobe and several smaller lesions in the left parietal lobe and cerebellum which indicated metastasis.

**Diagnosis:**

According to the history,physical examination and the radiological finding, the patient was diagnosed with malignant melanoma of central never system possibly.

**Interventions:**

The patient underwent left temporal and parietal lesions total resection with a craniotomy.

**Outcomes:**

The diagnosis of PDLM was established according to pathological characteristics and the negative finding of positron emission tomography (PET)-computed tomography (CT) outside CNS. The patient got no further treatment for economic reasons and experienced the progression and died 5 months after operation.

**Lessons:**

PDLM is extremely rare in CNS, as the clinical manifestation, radiological changes are not special, early diagnosis is difficult. The confirmed diagnosis is established by leptomeningeal biospy or surgical tissue. PET-CT can help differential diagnosis with metastastic leptomeningeal melanomas. The prognosis is dismal due to the inefficiency of chemotherapy or radiotherapy.

## 1 Introduction

Melanocytic neoplasms of the central nervous system (CNS) can be divided into primary neoplasms and metastasis. Importantly, primary melanocytic tumors are rare, and just account for only 1% of all melanomas and 0.05% of primary brain tumors.^[1]^ Lesions that are diffusely invasive in the leptomeninges, without evidence of extracranial metastasis, are categorized as primary diffuse leptomeningeal melanomatosis (PDLM).^[2]^ PDLM is a rare subtype of malignant melanomas of the CNS, with an incidence of 1 case per 10 million individuals.^[3]^ As the incidence is extremely low and the clinical manifestation of PDLM is not characteristic, it is often misdiagnosed as tubercular meningitis and hemorrhage. To the best of our knowledge, only 32 cases of PDLM have ever been reported in the literature, and only 4 of these were pediatric cases. Here we present a rare case of pediatric PDLM mimicking hemorrhage, which represents the very first pediatric PDLM case in China. In addition, we performed a comprehensive review of the related literature.

## 2 Case report

**Figure 1:**
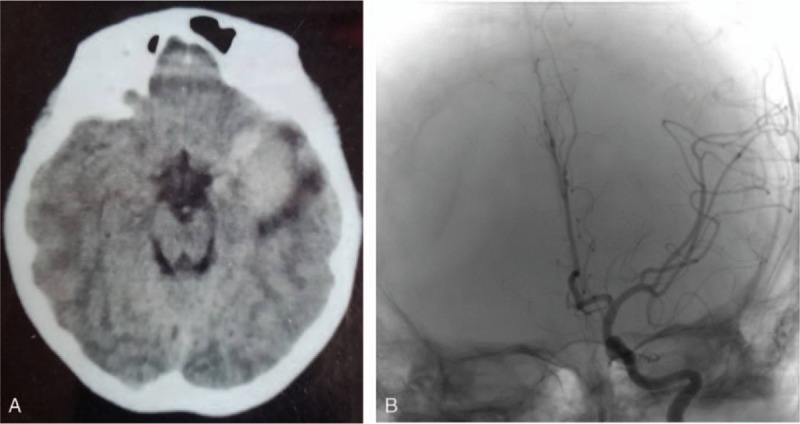
(A): CT scan revealing a high-density lesion in the left temporal lobe. (B): DSA showing negative findings.

**Figure 2:**
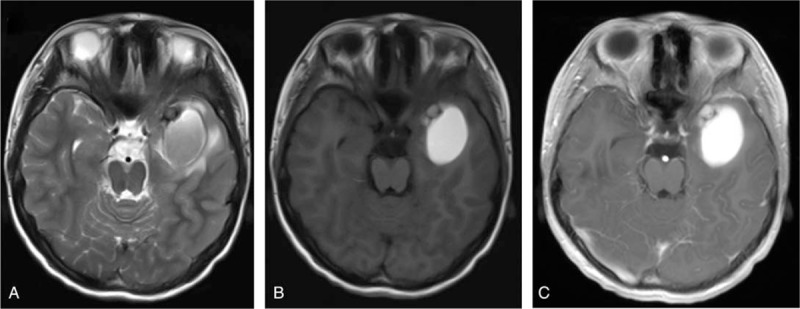
MRI scan of the lesion in the left temporal lobe with (A) iso-signals on T_2_-WI, (B) high signals on T_1_-WI, and (C) significant enhancement after administration of gadolinium.

**Figure 3:**
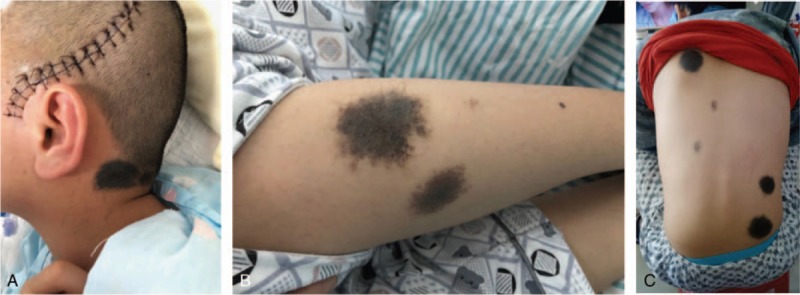
Physical examination showing multiple melanin changes in the skin of the whole body (A–C).

**Figure 4:**
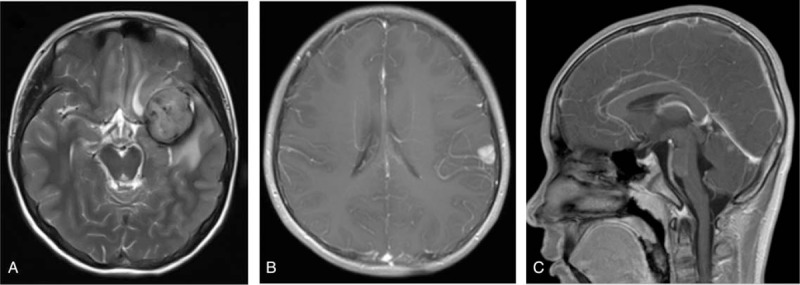
The second MRI scan showing significant enlargement of the lesion in the left temporal lobe (A), and metastatic lesions in the left parietal lobe (B) and the cerebellum (C).

**Figure 5:**
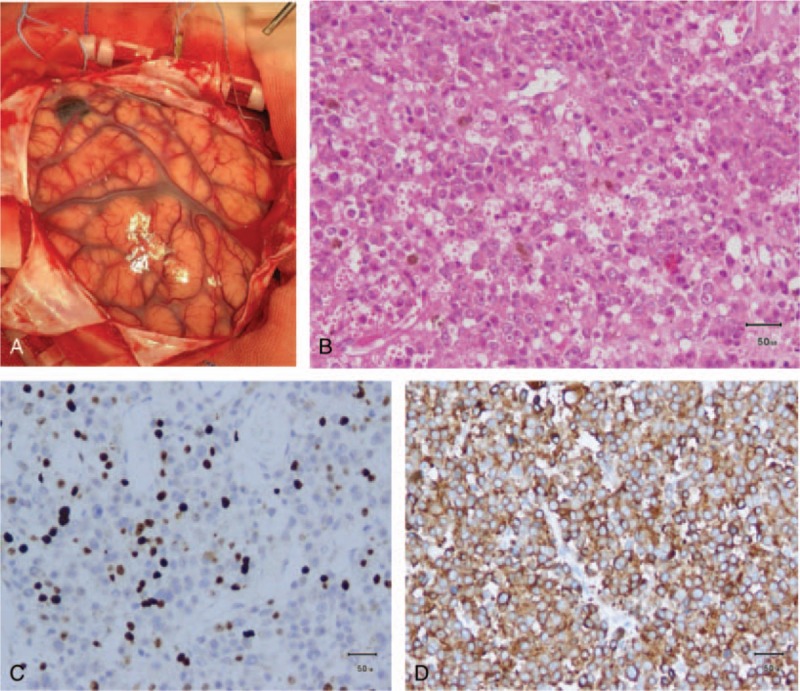
(A) Diffuse leptomeningeal thickness and melanin deposition observed intraoperatively. Pathological examination showing proliferation of tumor cells with melanin deposition in the cytoplasm (B), which were positive for Malen A (C) and HMB45 (D).

A 13-year-old boy was admitted to our department with symptoms that were described as “recurrent headache with vomiting for 5 months, and aggravating during the last week”. The patient presented with repeated headaches with no obvious triggers when presenting to our emergency department. A physical examination was negative and only showed cervical resistance. A brain computed tomography (CT) scan revealed a high-density lesion in the left temporal lobe, suggesting hemorrhage (Fig. 1A). No vascular abnormality was seen on digital subtraction angiography (DSA) (Fig. 1B). Magnetic resonance imaging (MRI) showed occupying lesions in the left temporal lobe, with high signals on T_1_-WI and isosignals on T_2_-WI, and with significant enhancement after administration of gadolinium (Fig. 2A-C). A diagnosis of spontaneous hemorrhage was made and conservative management was started. The patient was discharged after his headache was alleviated, but he returned 3 months later with a worse headache and reported vomiting again for 1 week. A comprehensive physical examination performed at that time showed multiple melanin changes in the skin of the whole body (Fig. 3A-C), which had been overlooked during the first hospitalization. MRI showed the significantly enlarged lesions in the left temporal lobe, with obvious surrounding edema, and several smaller lesions in the left parietal lobe and cerebellum, which indicated metastasis. This time, a diffuse leptomeningeal enhancement with both supratentorial and infratentorial areas was seen on administration of gadolinium (Fig. 4). A positron emission tomography (PET)-CT scan showed multiple active metabolic lesions in the brain but no skin melanin lesions outside the CNS. The patient underwent left temporal and parietal total lesion resection with craniotomy. Diffuse leptomeningeal thickness and melanin deposition were found intraoperatively (Fig. 5A). Postoperative pathological examination showed proliferation of tumor cells with melanin deposition in the cytoplasm that were positive for Malen A and HMB45 (Fig. 5B–D). A diagnosis of PDLM was established according to the pathological characteristics and the negative findings outside the CNS. Follow-up brain MRI showed that the left temporal and parietal lobe lesions had been totally removed. Over the next 5 months, the patient experienced progression but underwent no further treatment for economic reasons, and died 5 months after the operation.

## 3 Literature review and discussion

Melanomas are malignant tumors of the CNS, and can be divided into primary tumors and metastasis. Primary intracranial malignant melanomas, derived from the melanocytes of the leptomeninges,^[4]^ are rare, representing only 0.05% of all primary brain tumors.^[1]^ PDLM, recognized by its characteristic diffuse invasiveness of the leptomeninges with or without nodules formation^[5]^ is extremely rare, especially in pediatric patients. To date, only 4 pediatric cases have been described in the English-speaking literature (Table 1).^[4,6–8]^ Here we present a 13-year-old boy, as the fifth case, and provide a comprehensive review of clinical manifestations, differential diagnoses, and radiographic characteristics of PDLM.

**Table 1:**
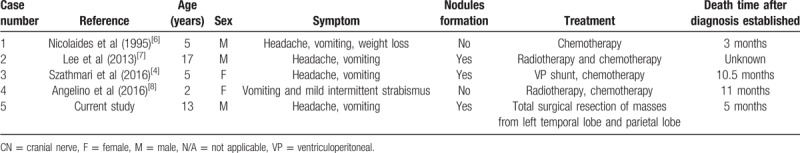
Literature review of all cases of PDLM.

The clinical manifestations of PDLM are nonspecific, mainly including headache, vomiting, and seizures, which often mimic the diagnosis of meningitis.^[3]^ Symptom progression depends on the location, size, and development speed of the lesions.^[9]^ Hydrocephalus may occur in the early stage due to leptomeningeal dissemination, leading to a disruption of cerebral spinal fluid (CSF) absorption. However, focal neurological deficits are rare. Considering that tumor apoplexy is common, the misdiagnosis of cerebral hemorrhage usually happens in the early stage such as in our case. Magnetic resonance angiography (MRA) or DSA can help eliminate bleeding due to vascular diseases. It has been reported that the proportion of patients with a large congenital nevus combined with intracranial malignant melanoma is about 25%.^[10]^ Hence, additional brain imaging must be performed to rule out the possibility of malignant melanoma if a patient suffers from increased intracranial pressure in combination with a large congenital melanocytic nevus.^[4]^

Imaging of PDLM also shows a lack of characteristic changes. Brain CT scans usually show a circular lesion with high-density mimicking hemorrhage. MRI scans may provide more diagnostic information of this rare disease. Due to the paramagnetic nature of melanin, typical MRI findings of PDLM may be high signals on T_1_-WI, iso-signals on T_2_-WI, and obvious enhancements after the injection of gadolinium.^[11]^ MRI enhanced scans may therefore help the diagnosis of this rare disease. Similarly, in the case reported here, the misdiagnosis was made based on imaging findings. Considering the vague presentation of symptoms and radiographic appearance of PDLM, a differential diagnosis should be considered in such cases. Considerations can include but are not limited to tubercular meningitis, hemorrhage, and metastatic melanomas. It has been reported that cerebral spinal fluid (CSF) analysis to confirm the pleomorphic cells with intracellular melanin and a high mitotic index can help an early diagnosis, although the positive rate is extremely low. PET-CT can support a differential diagnosis with metastastic leptomeningeal melanomas, as our case suggests.^[7]^

The confirmed diagnosis of PDLM depends on pathological examination, either leptomeningeal or surgical tissue biopsy. However, there is no significant difference to the general and histological changes observed in other melanocytic diseases.^[8]^ Identification requires immunohistochemic examination. The gross specimens of PDLM are mostly solid, growing on the surface of the brain, and are in close relation with the leptomeninges, with or without nodule formation. Intratumoral hemorrhage is common due to the rapid growth. Under the microscope, mixed epithelioid, and spindle tumor cells can be identified, displaying hyperchromatic nuclei. The melanin particles are visible, and the mitosis and abnormality are obvious. Immunohistochemical examinations show S-100 (+), HMB-45 (+), Melan A (+), and EMA (-);^[5]^ these changes are in line with the pathology observed in our case. PET-CT showed no changes outside the brain, and PDLM was thus diagnosed pathologically in combination with the observed clinical manifestations.

As PDLM is neither sensitive to chemotherapy nor radiotherapy, the prognosis is extremely poor, and the majority of patients die between 1 month and 2 years after onset, with an median survival time of 7 months.^[12]^ The cause of death is the progressive neurological deterioration and intractable intracranial hypertension. Recently, new molecular targeted drugs, such as the BRAF inhibitor vemurafenib and the antiprogrammed cell death-1 antibody nivolumab, have been reported to be effective against previously untreated metastatic melanoma.^[13,14]^ These studies show the potential for vemurafenib and nivolumab in the treatment of brain metastasis. The first clinical application of vemurafenib and nivolumab for PDLM has been reported by Fujimori and colleagues;^[15]^ however, whether this approach is useful for improving the prognosis needs to be assessed in a further clinical trial.

Herein, we present an extremely rare case of PDLM in a pediatric patient. The clinical manifestation and radiographic changes are not specific, and an early diagnosis is therefore difficult. The confirmed diagnosis is established on the basis of leptomeningeal or surgical tissue biopsy. PET-CT can help the differential diagnosis by identifying metastastic leptomeningeal melanomas. The prognosis is however dismal due to the inefficiency of chemotherapy and radiotherapy. To the best of our knowledge, this is the first pediatric case of PDLM in China.

## Author contributions

**Conceptualization:** Xinke Xu

**Data curation:** Xinke Xu, Yaqi Zheng

**Resources:** Fenghua Wang

**Investigation:** Junliang Li

**Writing – original draft:** Xinke Xu

**Writing – review and editing:** Xinke Xu, Fangcheng Li
